# Moderation of the Stressor-Strain Process in Interns by Heart Rate Variability Measured With a Wearable and Smartphone App: Within-Subject Design Using Continuous Monitoring

**DOI:** 10.2196/28731

**Published:** 2021-10-04

**Authors:** Herman de Vries, Wim Kamphuis, Hilbrand Oldenhuis, Cees van der Schans, Robbert Sanderman

**Affiliations:** 1 Professorship Personalized Digital Health Hanze University of Applied Sciences Groningen Netherlands; 2 Department of Human Behaviour & Training TNO Soesterberg Netherlands; 3 Department of Health Psychology University Medical Center Groningen Groningen Netherlands; 4 Department of Rehabilitation Medicine University Medical Center Groningen Groningen Netherlands; 5 Research Group Healthy Ageing Allied Health Care and Nursing Hanze University of Applied Sciences Groningen Netherlands; 6 Department of Psychology Health and Technology University of Twente Enschede Netherlands

**Keywords:** stress, strain, burnout, resilience, heart rate variability, sleep, wearables, digital health, sensors, ecological momentary assessment, mobile phone

## Abstract

**Background:**

The emergence of smartphones and wearable sensor technologies enables easy and unobtrusive monitoring of physiological and psychological data related to an individual’s resilience. Heart rate variability (HRV) is a promising biomarker for resilience based on between-subject population studies, but observational studies that apply a within-subject design and use wearable sensors in order to observe HRV in a naturalistic real-life context are needed.

**Objective:**

This study aims to explore whether resting HRV and total sleep time (TST) are indicative and predictive of the within-day accumulation of the negative consequences of stress and mental exhaustion. The tested hypotheses are that demands are positively associated with stress and resting HRV buffers against this association, stress is positively associated with mental exhaustion and resting HRV buffers against this association, stress negatively impacts subsequent-night TST, and previous-evening mental exhaustion negatively impacts resting HRV, while previous-night TST buffers against this association.

**Methods:**

In total, 26 interns used consumer-available wearables (Fitbit Charge 2 and Polar H7), a consumer-available smartphone app (Elite HRV), and an ecological momentary assessment smartphone app to collect resilience-related data on resting HRV, TST, and perceived demands, stress, and mental exhaustion on a daily basis for 15 weeks.

**Results:**

Multiple linear regression analysis of within-subject standardized data collected on 2379 unique person-days showed that having a high resting HRV buffered against the positive association between demands and stress (hypothesis 1) and between stress and mental exhaustion (hypothesis 2). Stress did not affect TST (hypothesis 3). Finally, mental exhaustion negatively predicted resting HRV in the subsequent morning but TST did not buffer against this (hypothesis 4).

**Conclusions:**

To our knowledge, this study provides first evidence that having a low within-subject resting HRV may be both indicative and predictive of the short-term accumulation of the negative effects of stress and mental exhaustion, potentially forming a negative feedback loop. If these findings can be replicated and expanded upon in future studies, they may contribute to the development of automated resilience interventions that monitor daily resting HRV and aim to provide users with an early warning signal when a negative feedback loop forms, to prevent the negative impact of stress on long-term health outcomes.

## Introduction

### Background

Psychological stress is associated with increased risk of several forms of cancer [1], musculoskeletal diseases [2], periodontal diseases [3], type 2 diabetes mellitus [4], stroke [5], cardiovascular disease [6], and recurrent cardiovascular disease [7]. In an occupational setting, psychosocial risk factors such as high job demands are estimated to increase the risk of stress-related diseases (eg, burnout) by 60%-90% [8]. Occupational stress can therefore cause absenteeism, organizational dysfunction, and decreased productivity, and it has a large economic burden [9].

Stress occurs when the brain subconsciously appraises a demand as threatening because of a lack of resources to cope with it [10]. This threat appraisal that we refer to as stress is sometimes referred to as *distress*, whereas demands for which sufficient coping resources are available are appraised as a challenge or as *eustress*. Therefore, stress can be seen as a psychological state that is the result of a divergence between demands on an individual and the individual’s perceived capacity to cope with them. Stress causes an imbalance in the body’s biological equilibrium (homeostasis), which requires a neural, neuroendocrine, and neuroendocrine-immune adaptation to restore it (allostasis) [11,12]. Although acute stress can have negative effects, it is particularly the cumulative wear and tear on bodily systems (allostatic load) caused by excessive stress or inefficient management of the systems that promote adaptation that is detrimental to long-term health and well-being [13]. In addition, lifestyle-related factors such as obesity, sleep, and substance abuse can also contribute to allostatic load [14]. Allostatic load is therefore considered a measure of the cumulative biological burden on health [15].

To complement this biological and neuroendocrinological perspective on the negative long-term health effects of stress and provide a framework for how short-term spillover effects of stress accumulate the need for a recovery concept be introduced [16]. A need for recovery arises when an individual has problems using resources to adaptively cope with demands that induce stress [17]. Need for recovery is a conscious emotional state that is related to the temporal depletion of resources following effort to meet demands and is characterized by feelings of mental exhaustion [18]. As the availability of resources is assessed during appraisal and the use of resources may be needed during coping, the Conservation of Resources Theory states that an initial loss of resources can lead to a negative feedback loop that increases one's vulnerability to stress [19]. Such a loss spiral may become even more distinct if stress negatively impacts the recovery process itself, for instance, by negatively impacting sleep quality [20] and psychological detachment [21]. Resilience, which can be defined as the process of positively adapting to adverse events [22], is a term describing this process from a positive perspective. During a resilient process, the aforementioned loss spiral is prevented by using resources to adaptively cope with demands and stress to limit long-term strain and its related negative consequences on health and well-being from developing [23]. Resilience is therefore an ongoing process that influences the extent to which adverse events that occur on a small timescale have an impact on mid- to long-term health outcomes.

### Heart Rate Variability

**Figure 1:**
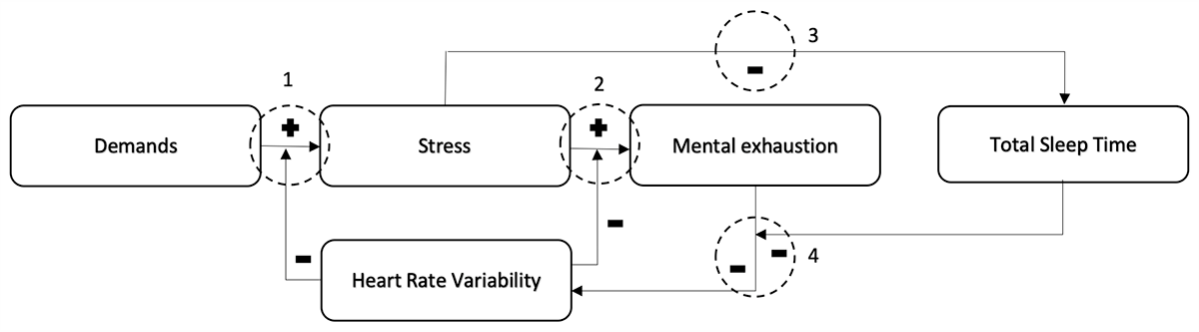
The conceptual model for this study and the four hypotheses that will be tested.

A challenge for resilience research that focuses on resilience-related associations on timescales is that it requires continuous data collection, making it relatively labor-intensive for participants to do so. Over the past decade, the emergence of smartphones and wearable sensor technologies has enabled the easy and unobtrusive measurement of physiological and psychological data related to an individual’s resilience [24]. A promising example of such a metric is heart rate variability (HRV), which refers to the variation in interbeat intervals of the heartbeats [25]. HRV is a plausible, noninvasive, and easily applicable biomarker for resilience that may serve as a global index of an individual’s flexibility and adaptability to stressors [26,27]. HRV is negatively correlated with allostatic load, illustrating its use as an overall health risk indicator [28]. Stress is also known to decrease HRV, particularly with reduced parasympathetic activation [29-31]. Although an acute decline in HRV may be indicative of increased acute stress levels, HRV can remain lowered during rest and sleep after stress or mental exhaustion [32-35]. Conversely, having a lower trait resting HRV has been linked to increased sensitivity to stress via appraisal when faced with demands [36] and to suboptimal emotion regulation that may result in mental exhaustion [37,38]. Therefore, resting HRV can be seen as a physiological resource that is addressed during the appraisal of demands and coping with stress. Therefore, resting HRV can be hypothesized to have a buffering effect on the positive associations between demands and stress, as well as between stress and mental exhaustion. These two hypothesized buffering effects are depicted as circles 1 and 2 in Figure 1, which represent the conceptual model for this study and were based on a previous publication [39]. The model is based on the Transactional Model of Stress and Coping [10], the Job Demands-Resources Model of Burnout [40], the Effort-Recovery Model [17], and the Conservation of Resources Theory [19]. In short, it depicts that demands are appraised as stress when resources are low, that stress leads to mental exhaustion when resources to cope with the demands are lacking, and that mental exhaustion limits resources to deal with future demands, unless there are sufficient recovery opportunities. In this study, HRV is the resource of interest, whereas sleep, operationalized as total sleep time (TST), represents the model’s recovery process.

### Sleep

Besides resting HRV, sleep is also a relevant potential indicator for the accumulation of the negative consequences of stress and predictor of spillover need for recovery. In the literature, stress has been consistently shown to decrease slow-wave sleep, rapid eye movement sleep, and sleep efficiency, as well as to increase the number of awakenings that may impact the overall sleep duration [20]. Therefore, stress can be hypothesized to negatively affect the TST, which is the total time during a sleep episode in which one was not awake (Figure 1; hypothesis 3). In contrast, sleep has important homeostatic functions that are essential during recovery from both physiological and psychological strains [41]. Sleep deprivation therefore has been linked to an increase in allostatic load [42] and has been linked to decreased HRV in some studies [43,44]. As mental exhaustion may result in decreased resting HRV [34,35] and sleep is an essential aspect of the recovery process, TST can be hypothesized to buffer against the negative association between mental exhaustion and resting HRV (Figure 1; hypothesis 4).

### Aims of This Study

HRV measurement is regularly used as a biofeedback tool in mobile health (mHealth) interventions that target acute stress relief [45-47] but may also be useful for interventions that aim to provide users with feedback on their resilience over a longer timeframe. A recent literature review confirmed that HRV has potential as a biomarker for resilience but suggested that more longitudinal studies are needed that use wearable sensors to observe HRV in a naturalistic context of real-life and associate it with resilience-related outcomes, as most of the evidence is based on cross-sectional population studies [27]. Therefore, this study longitudinally assessed the aforementioned hypotheses in a free-living context using consumer-available wearable sensors. Exploring these will provide insight into the potential causal pathways of the within-day accumulation of the negative consequences of stress. Gained insights may therefore be beneficial to the future development of (automated) resilience interventions that target the prevention of stress-related health problems.

## Methods

The study protocol was approved by the ethical committee of the Hanze University of Applied Sciences Groningen (heac.2018.008) in the Netherlands.

### Participants

Students in applied psychology, social work, and physiotherapy who were about to start their first full-time internship were invited to participate via a message on the school’s digital learning environment and email. This population was anticipated to be at risk of experiencing stress because of the potentially stressful nature of these internships, as well as the fact that this was the first internship in the participants’ curriculum. A maximum of 15 participants could be simultaneously recruited because of the availability of materials. The recruitment and data collection processes were therefore divided into two waves that started in September 2018 and September 2019, respectively. The students were sent an email with an information letter that described the goal of the study, a description of the measurement protocol, and management of the collected data; the email also stated that participation would be unrelated to their internship or educational progress, that participation would occur on a voluntary basis, and that they could stop at any time without negative consequences. Some of the researchers were employed by the university in which the students were enrolled but had no other associations with the invited students (eg, via education). The participants provided written informed consent before participation. Participants who collected complete data on at least 84 days during the formal participation period were rewarded with a €25 (US $27.50) gift voucher to facilitate recruitment and optimize adherence during participation. This reward threshold represents an adherence of at least 80% over a data collection period of 15 weeks (105 days). The threshold was solely used as a cutoff point for the reward and not for statistical analyses.

### Data Collection

Participants were assisted in installing the required apps on their smartphones and were instructed on how to use the devices used for data collection. The data collection period started immediately after the measurement instructions, after which participants collected data for 15 weeks. Some participants completed additional daily measurements on a voluntary basis until their appointment was planned to return the used materials. At this appointment, an additional 20-minute conversation was held about how they experienced the daily measurements and to learn about potential improvements for future studies. During the study, anonymized user accounts were used for the applied consumer-available wearables in order to protect the participants’ privacy on the companies’ cloud servers, before being exported and deleted by the researchers after completing their participation.

#### Main Variables

*Resting HRV* was measured daily using a consumer-available Polar H7 Bluetooth chest strap in combination with the Elite HRV app that is freely available in the iOS App Store and Google Play Store. The Polar H7 chest strap has been shown to accurately measure resting HRV when compared with an electrocardiogram [48]. The Elite HRV app was chosen because it is easy to use for daily HRV measurements in a consumer setting and allows data export on an interbeat interval level. Participants were instructed to perform a 2-minute HRV measurement in a supine position after awakening before getting out of bed. This is consistent with existing standards and recommendations that suggest a measurement duration of 1-5 minutes and consistent circumstances with as little influence as possible from circadian rhythms, meals, smoking, posture changes, and before significant mental or physical exertion [49,50]. We considered sitting or standing resting HRV measurements to account for the possible presence of parasympathetic saturation in case we recruited an elite endurance athlete [51]. However, we opted for supine measurements immediately after awakening in order to limit the potential influence of posture changes, physical activity, meals, and smoking as recommended by the aforementioned guidelines, as well as to ensure that all participants performed the measurement at a similar postawakening time and in a similar context.

The wrist-worn Fitbit Charge 2 activity tracker was used to measure *TST*, which tends to slightly overestimate but for which has acceptable measurement accuracy in diverse populations [52-54]. Participants were instructed to continuously wear the Fitbit during the day and night and charge it at least once every 5 days.

Before bedtime (available between 08 PM and 06 AM), participants completed a short ecological momentary assessment (EMA) questionnaire using an internally developed smartphone app to measure *demands*, *stress*, and *mental exhaustion*. The daily EMA questionnaire data were stored on premise. In the absence of a single item or full scale that was relevant for the study setting, demands were scored on the self-composed diary question, “How demanding was your day?” These demands represent the contextual circumstances that exerted pressure on the participant, whereas stress reflected the resulting threat appraisal that these evoked within the individual. Stress was scored on a validated single-item scale [55]: “How much stress did you perceive today?” Mental exhaustion is an aspect of the need for recovery concept and was based on item 3 of the Need for Recovery Scale [56], which was chosen because it appropriately represents strain within the context of the used conceptual model [39]: “I felt mentally exhausted as a result of my activities.” All three items were scored on a 11-point numeric rating scale ranging from 0 (*not at all* for demands and stress and *strongly disagree* for mental exhaustion) to 10 (*extremely* for demands and stress and *strongly agree* for mental exhaustion).

#### Control Variables

Although the Fitbit Charge 2 was chosen for its accuracy in measuring TST, its data on *moderate-to-vigorous physical activity (MVPA)* and *sedentary time* were also used during analysis as potential confounders. MVPA is defined as the total amount of daily minutes where the participant was physically active at an intensity of 3 metabolic equivalents or more, where 1 metabolic equivalent represents the resting metabolism. In previous studies, MVPA was negatively associated with state anxiety [57], mental strain [58], and HRV recovery [59], as well as positively associated with TST [60]. Similarly, sedentary time was positively associated with depression and anxiety [61] and negatively associated with TST [62] and HRV [63]. Finally, Fitbit-measured TST was also used as a control variable in the analyses for stress and mental exhaustion because intraindividual variability in accelerometer-measured TST has been associated with increased stress [64].

In addition, *alcohol consumption* during the previous day was measured in a morning questionnaire (available between 6 AM and 3 PM) for use as a potential confounder. In previous literature, alcohol consumption has been negatively associated with wearable-measured TST [65] and reduced HRV [66]. Alcohol consumption was scored as a numeric variable by asking for the number of alcoholic beverages consumed during the previous day. Although the absolute amount of alcohol in different types of beverages may deviate, asking for the number of alcoholic beverages consumed is both convenient for daily inquiry and consistent with the widely used AUDIT-C questionnaire [67].

### Data Analysis

All data management and analyses were performed in RStudio [68] and R [69].

#### Data Management

For HRV data management, the RHRV package [70] was used. Interbeat interval data of all daily observations were filtered for artifacts using the algorithm in the RHRV package. The respective algorithm is described fully in a complementary book written by the authors of the RHRV package [71]. The algorithm is too comprehensive to be fully described here but is summarized by the authors to apply an adaptive threshold to reject beats whose interbeat interval value differs from previous and following beats, and from a mobile mean more than a threshold value, as well as beats that are not within acceptable physiological values. Subsequently, the root mean square of the successive differences (RMSSD) was calculated for every observation by first calculating each successive difference between heartbeats in milliseconds, then squaring these values, averaging that result, and finally taking its square root [72]. However, algorithmic artifact correction can only distinguish potential measurement errors on an interbeat interval level and can result in abnormally high RMSSD values if there are too many measurement errors present. As this study was performed in free-living conditions, it was not possible to verify if participants performed the daily measurements exactly as instructed. Therefore, a second filtering method was applied to filter out HRV observations with extreme RMSSD values for that specific participant. To achieve this, within-subject RMSSD outliers of daily observations with a value that lies more than 1.5 IQR below the first quartile or 1.5 IQR above the third quartile for all available data of the respective participant were removed [73]. Finally, the RMSSD values were logarithmically transformed to improve the distribution for parametric statistical modeling of resting HRV.

The TST data were filtered for episodes that started after filling in the evening EMA questionnaire and ended before the morning questionnaire was filled in to obtain the nighttime TST. When more than one TST episode was present between the evening and the subsequent morning EMA questionnaire, they were combined. No outliers were removed from the EMA data because no unfeasible values were identified.

Because of the different scales of the resting HRV, TST, and EMA data, centering and standardizing the data was necessary to prevent potential multicollinearity and allow comparability of the coefficients of the independent variables. As the level 1 association between the aforementioned main variables is the primary interest in this study, centering within subject is recommended as opposed to centering at the grand mean [74]. Therefore, all data were centered and standardized within subjects by subtracting the subject’s mean value over all daily observations from each value and dividing it by the subject’s SD over all daily observations. The z-scores that were used during analysis therefore reflect the degree to which a daily observation differed from the individual’s own mean. As the mean z-scores for each variable in each individual were zero, there was no between-subject variance left in the data. Therefore, multiple regression analysis was performed instead of the multi-level modeling that we originally planned to undertake, despite the observations being nested within subjects (Linear Mixed Modeling with fixed effects and random slopes using the within-subject standardized values resulted in the same outcomes and conclusions on all analyses but had a boundary [singular] fit and no differences in the within- and between-subject explained variance because there was no between-subject variance. As these multi-level models had no benefit, the results of our multiple regression analyses were presented in this study).

#### Statistical Analysis

To test the four hypotheses described in the Introduction, four statistical analyses were performed. In the first analysis, stress was first modeled based on the control variables MVPA, sedentary time, and previous-day TST, after which the main variables demands, resting HRV, and the interaction effect of demands and resting HRV were added to create the full model. In the second analysis, a control variable model for mental exhaustion was first developed based on MVPA, sedentary time, and previous-night TST, after which a full model was created by adding the main effects of stress and resting HRV, as well as the interaction effect between stress and resting HRV. For analysis three, the control variable model for TST contains previous-day MVPA, sedentary time, and alcohol consumption, with previous-day stress being added to the full model. Finally, the fourth analysis first modeled resting HRV based on control variables previous-day MVPA, sedentary time, and alcohol consumption before adding the main effects of previous-evening mental exhaustion and previous-night TST, as well as the interaction effect between previous-evening mental exhaustion and previous-night TST. To compare the explained variance and statistical significance of the control variable and full models, the difference in the adjusted R-squared value and F statistic was calculated for all four analyses.

## Results

### Overview

A total of 26 participants were recruited for this study. The participants were predominantly women (n=24). Most participants studied applied psychology (n=19), followed by social work (n=6) and physiotherapy (n=1). The participants were aged between 19.2 and 33.2 years (median 22.6). The participants collected TST data on 2129 days (per participant range 10-119; median 94), 1731 morning EMA questionnaires (range 5-109; median 74), 1653 evening EMA questionnaires (range 7-111; median 73), and HRV data on 1443 days (range 6-115; median 53). In total, for 1004 of the 2379 days (42.2%) on which a participant collected data, the participant collected complete data containing all required TST, HRV, and EMA data. The descriptive statistics for and intercorrelations between the main and control variables are presented in Table 1. Three participants did not complete the full (105 days) measurement period because they lost motivation for the daily measurements, and one participant stopped the daily measurements because of skin rash related to wearing the Fitbit. All three participants who did not complete the full measurement period contributed daily measurements and were thus still included in the analyses. During the exit conversations, several participants stated that they found it difficult to adhere to the HRV measurement, because the need to apply a moistened chest strap and lay still for 2 minutes after awakening while they wanted to continue with their day was inconvenient. Missing Fitbit data were primarily ascribed to forgetting to charge it, particularly when participants were away from home. Finally, participants mostly attributed missing EMA data to simply forget to act on the smartphone notification as they were busy at that time.

**Table 1:** Descriptive statistics for and intercorrelations between the main (1-5) and control (6-8) variables.

Variable			HRV^a^		TST^b^		Demands		Stress		Mental exhaustion		MVPA^c^		Sedentary time		Alcohol use		Value, mean (SD)	
**HRV**																				75.3 (49.9)
	Correlation	—^d^		—		—		—		—		—		—		—				
	*P* value	—		—		—		—		—		—		—		—				
**TST**																				7.3 (1.4)
	Correlation	−.03		—		—		—		—		—		—		—				
	*P* value	.36		—		—		—		—		—		—		—				
**Demands**																				4.6 (2.4)
	Correlation	.06		−.01		—		—		—		—		—		—				
	*P* value	.03		.68		—		—		—		—		—		—				
**Stress**																				3.6 (2.4)
	Correlation	.06		.02		.53		—		—		—		—		—				
	*P* value	.03		.42		<.001		—		—		—		—		—				
**Mental exhaustion**																				3.6 (2.4)
	Correlation	.004		−.03		.55		.64		—		—		—		—				
	*P* value	.89		.31		<.001		<.001		—		—		—		—				
**MVPA**																				33.9 (37.0)
	Correlation	−.05		−.11		.04		−.05		−.06		—		—		—				
	*P* value	.10		<.001		.13		.06		.02		—		—		—				
**Sedentary time**																				687.2 (220.7)
	Correlation	−.13		−.48		−.07		.06		.03		−.24		—		—				
	*P* value	<.001		<.001		.004		.02		.18		<.001		—		—				
**Alcohol use**																				1.3 (2.8)
	Correlation	−.04		−.28		−.21		−.10		−.06		.10		.08		—				
	*P* value	.13		<.001		<.001		<.001		.03		<.001		.002		—				

^a^HRV: heart rate variability (in milliseconds).

^b^TST: total sleep time (in hours).

^c^MVPA: moderate-to-vigorous physical activity (in minutes).

^d^Not applicable.

### Analysis 1: Stress

A two-step hierarchical multiple regression model explaining stress scores was developed (Table 2). After controlling for MVPA, sedentary time, and previous-night TST, demands were positively associated (*P*<.001) with stress. In addition, the interaction effect of demands and resting HRV significantly (*P*=.044) buffered against this positive association. This means that participants tended to report higher stress scores on days that they also considered to be more demanding, but this relationship was weaker on days where the participant woke up with a relatively high resting HRV. The positive association between demands and stress, as well as the buffering effect of resting HRV, confirms hypothesis one. Furthermore, the control variable MVPA was positively associated with stress (*P*=.044), which means that participants reported higher stress scores on days where they were more physically active. In the control variable model, TST was a negative predictor of stress (*P*=.03), but this effect was no longer significant in the full model. The control variable model of analysis explained 2.0% of the within-subject variance in the daily stress scores, whereas the full model had an explained variance of 21.7%, which is a significant increase from the control variable model.

**Table 2:** Hierarchical multiple regression model for stress (analysis 1).

Independent variable	Stress				
	Step 1 (n=953)^a^			Step 2 (n=953)^b^	
	β	*P* value	β		*P* value
Intercept	.05	.14	.00		.96
TST^c^	−.09	.03	.00		.89
MVPA^d^	.12	<.001	.06		.04
Sedentary time	.02	.75	.05		.29
Demands	—^e^	—	.47		<.001
HRV^f^	—	—	.01		.70
Demands×HRV	—	—	-.06		.04

^a^Adjusted R-squared 0.02; *F*_3,949_=7.541.

^b^Adjusted R-squared 0.22; *F*_6,946_=44.86; Δ adjusted R-squared 0.20; Δ *F*_6,949_=37.32.

^c^TST: total sleep time (in hours).

^d^MVPA: moderate-to-vigorous physical activity (in minutes).

^e^Variable is not included in step 1.

^f^HRV: heart rate variability (in milliseconds).

### Analysis 2: Mental Exhaustion

A two-step hierarchical multiple regression model explaining mental exhaustion scores was developed (Table 3). After controlling for MVPA, sedentary time, and previous-night TST, stress was positively associated (*P*<.001) with mental exhaustion. In addition, the interaction effect of stress and resting HRV significantly (*P*=.029) buffered against this positive association. This means that participants tended to report higher mental exhaustion scores on days that they were also considered stressful, but this relationship was weaker on days where the participant woke up with a relatively high resting HRV. The positive association between stress and mental exhaustion, as well as the buffering effect of resting HRV confirm hypothesis two. In the control variable model, MVPA was also positively associated with mental exhaustion (*P*=.017), but this effect was no longer significant in the full model. The control variable model of analysis two explains 1.4% of the within-subject variance in the daily mental exhaustion scores, whereas the full model has an explained variance of 31.6%, which is a significant increase from the control variable model.

**Table 3:** Multiple regression model for mental exhaustion (analysis 2).

Independent variable	Mental exhaustion				
	Step 1 (n=953)^a^			Step 2 (n=953)^b^	
	β	*P* value	β		*P* value
Intercept	.05	.12	.02		.37
TST^c^	−.06	.15	−.01		.68
MVPA^d^	.09	.02	.02		.58
Sedentary time	.09	.09	.07		.09
Stress	—^e^	—	.55		<.001
HRV^f^	—	—	−.04		.15
Stress × HRV	—	—	−.06		.03

^a^Adjusted R-squared 0.01; *F*_3,949_=5.42.

^b^Adjusted R-squared 0.32; *F*_6,946=_74.17; Δ adjusted R-squared 0.31; Δ *F*_6,946_=68.75.

^c^TST: total sleep time (in hours).

^d^MVPA: moderate-to-vigorous physical activity (in minutes).

^e^Variable is not included in step 1.

^f^HRV: heart rate variability (in milliseconds).

### Analysis 3: Total Sleep Time

A two-step hierarchical multiple regression model explaining nighttime TST was developed (Table 4). After controlling for previous-day MVPA, sedentary time, and alcohol consumption, previous-day stress did not predict TST, unlike our expectation based on hypothesis 3. However, the control variables previous-day MVPA and alcohol consumption were negatively associated with TST, whereas sedentary time was positively associated with TST. This means that participants had a lower TST on days where they were relatively physically active, consumed alcohol, and had limited sedentary time. The control variable model of analysis three explains 3.8% of the within-subject variance in TST, whereas the full model has an explained variance of 4.6%, which is not a statistically significant increase from the control variable model.

**Table 4:** Multiple regression model for TST (analysis 3).

Independent variable	TST^a^				
	Step 1 (n=1285)^b^			Step 2 (n=1285)^c^	
	β	*P* value	β		*P* value
Intercept	−.03	.32	−.03		.33
MVPA^d^	−.07	.01	−.07		.01
Sedentary time	.08	.01	.08		.01
Alcohol consumption	−.20	<.001	−.20		<.001
Stress	—^e^	—	−.01		.64

^a^TST: total sleep time (in hours).

^b^Adjusted R-squared 0.05; *F*_3,1281_=21.88.

^c^Adjusted R-squared 0.05; *F*_4,1280_=6.46; Δ adjusted R-squared 0.0; Δ *F*_4,1280_=−5.42.

^d^MVPA: moderate-to-vigorous physical activity (in minutes).

^e^Variable is not included in step 1.

### Analysis 4: Heart Rate Variability

A two-step hierarchical multiple regression model explaining resting HRV was developed (Table 5). After controlling for previous-day MVPA, sedentary time, and alcohol consumption, previous-evening mental exhaustion negatively predicted (*P*<.001) resting HRV, but previous-night TST did not buffer against this negative association. Therefore, these results partially support hypothesis four. Among the control variables, previous-day alcohol consumption negatively predicted resting HRV (*P*<.001). The control variable model explained 2.3% of the within-subject variance in resting HRV, whereas the full model had an explained variance of 3.6%, which was not a statistically significant increase from the control variable model.

**Table 5:** Multiple regression model for HRV (analysis 4).

Independent variable	HRV^a^				
	Step 1 (n=948)^b^			Step 2 (n=948)^c^	
	β	*P* value	β		*P* value
Intercept	−.00	.98	.00		.96
MVPA^d^	−.06	.06	−.05		.10
Sedentary time	.02	.53	.03		.40
Alcohol consumption	−.18	<.001	−.19		<.001
Mental exhaustion	—^e^	—	−.12		<.001
TST^f^	—	—	.02		.52
Mental exhaustion × TST	—	—	−.00		.92

^a^HRV: heart rate variability (in milliseconds).

^b^Adjusted R-squared 0.02; *F*_3,944_=8.42.

^c^Adjusted R-squared 0.04; *F*_6,941_=6.956; Δ adjusted R-squared 0.02; Δ *F*_6,941_=−1.46.

^d^MVPA: moderate-to-vigorous physical activity (in minutes).

^e^Variable is not included in step 1.

^f^TST: total sleep time (in hours).

## Discussion

### Principal Findings

This study aimed to test the hypotheses that (1) demands are positively associated with stress and resting HRV buffers against this association, (2) stress is positively associated with mental exhaustion and resting HRV buffers against this association, (3) stress negatively impacts subsequent-night TST, and (4) previous-evening mental exhaustion negatively impacts resting HRV, while previous-night TST buffers against this association. By assessing these associations based on longitudinal data that were collected using consumer-available wearables and smartphone apps in a free-living context, this study provides insight into the potential pathways of the within-day accumulation of the negative consequences of stress. The results of this study support hypotheses one and two and partially support hypothesis four.

### Heart Rate Variability as an Index of Resilience

As hypothesized, having a high resting HRV buffered against the positive associations between demands and stress (hypothesis 1), as well as between stress and mental exhaustion (hypothesis 2). Similarly, mental exhaustion negatively predicted resting HRV, as expected (hypothesis 4). These findings suggest that waking up with a relatively high intraindividual resting HRV decreases an individual’s sensitivity to stress when faced with demands, as well as the likelihood of being mentally exhausted during a stressful day. In addition, as the accumulation of mental exhaustion negatively impacts an individual’s resting HRV, an increase in mental exhaustion negatively impacts this (psycho) physiological resource and thus potentially creates a negative feedback loop that can lead to a loss spiral. These results therefore confirm our hypothesis that a decline in resting HRV is indicative of the accumulation of the negative consequences of stress, as well as the continued accumulation of negative consequences of stress. Therefore, resting HRV can be seen as a biomarker for or an index of resilience, where a decline in resting HRV may signal that buildup of allostatic load is present and suggests that the individual’s readiness to face new demands may at least be temporarily decreased.

As highlighted in a recent literature review, most studies to date investigating the role of HRV as an index for resilience have a cross-sectional nature and assess relationships at the between-subject level [27]. To our knowledge, this study is the first to apply a nested longitudinal design and assess the potential of resting HRV as an index of resilience to stress on a within-subject level. Previous studies that investigated between-subject differences identified similar relationships between resting HRV and mental health outcomes. For instance, a recent study with school teachers concluded that 48-hour trait HRV buffered the effect of emotional demands on exhaustion [75]. Another recent study cross-sectionally assessed a population of young female adults and found that having a high resting HRV buffered against the positive association between emotion regulation difficulties and depressive symptoms [76]. Resting HRV has also been reported to buffer against the negative effects of chronic stress on sleep quality, which in turn is related to greater depressive symptoms [77]. Finally, high stress-induced HRV was shown to buffer against the negative effect of hostility on cortisol sensitivity [78]. Therefore, the within-subject findings of this study align with previous studies that also reported favorable between-subject effects of resting HRV on diverse mental health outcomes.

### The Role of Sleep in the Within-Day Stressor-Strain Process

Contrary to our hypothesis, stress did not negatively affect TST (hypothesis 3). The absence of a negative association between stress and TST conflicts with previous literature that consistently links experimental stress to decreased slow-wave sleep, rapid eye movement sleep, sleep efficiency, and increases in awakenings [20]. A possible explanation for this could be the difference in context, as those studies examined the influence of experimental stress on polysomnographically measured sleep, whereas this study investigated daytime stressors and TST in a natural free-living context. Because of the increasing capabilities and performance of consumer wearables to measure sleep and the resulting rise in the use of consumer wearables in sleep research, future studies may increase insights into the potential relationship between daily stressors and TST in a natural free-living context [79].

TST also did not buffer against the negative association between mental exhaustion and resting HRV, as expected (hypothesis 4). This expectation was based on the rationale that sleep has important homeostatic functions that are essential during recovery from strain [41], and that sleep deprivation has been linked to an increase in allostatic load [42] and a decrease in HRV [43,44]. As mental exhaustion was measured during the evening and resting HRV during the morning, we expected TST to potentially have a buffering effect, meaning that the negative impact of mental exhaustion on resting HRV would be smaller if the participant slept well that night. This buffering effect was not present in these findings, but TST was also not positively associated with resting HRV, as might be expected based on the aforementioned literature. A possible explanation for this could be that the relationship between sleep deprivation and HRV in previous literature seems to be particularly present in studies assessing a longer sleep deprivation period [80], which might suggest that the nuanced day-to-day differences in TST are too small to significantly impact the resting HRV. Future studies investigating the impact of TST on resting HRV or the recovery from strain in a natural free-living context in which such long periods of sleep deprivation are relatively uncommon, assessing the impact of multi-day trends in TST might help increase insight on this topic.

### Notable Effects of MVPA, Sedentary Time, and Alcohol Consumption

The effects of most of the control variables that were significantly associated with the outcomes of the four analyses were as expected, but some of the effects seem to conflict with previous literature. For instance, MVPA was negatively associated with TST, but a recent study found a positive association between MVPA and TST [60]. Similarly, sedentary time was positively associated with TST in this study, whereas a negative association with TST was reported in another recent study assessing obese adults [62]. A possible explanation can be found in the reported significant correlations between MVPA, sedentary time, and alcohol consumption (Table 1). This young student population spends part of their leisure time enjoying the local nightlife, in which dancing and alcohol consumption are common. It is therefore possible that this will have caused the low sedentary time, high MVPA, and alcohol consumption that were associated with low TST.

### Strengths and Limitations

The strengths of this study are its longitudinal design and large sample of nested observations, optimizing the within-subject variance. Moreover, the use of consumer-friendly wearable sensors and smartphone apps allowed for relatively unobtrusive monitoring in a free daily living context, optimizing the generalizability for similar settings. A limitation of this study was the need to apply relatively coarse algorithmic artifact correction and rule-based outlier filtering during HRV data management. Because of the choice to use a consumer-available sensor and app for long-term daily measurements in free-living conditions, electrogram-level data were unavailable, and it was impossible to verify if participants performed the measurements as instructed. As the applied algorithmic artifact removal method can only filter out interbeat interval artifacts within an HRV measurement, it has no rules to decide whether an observation should be removed altogether, filtering out extreme within-subject RMSSD outliers was necessary. Furthermore, algorithm-based artifact correction was preferred over manually adjusting interbeat interval artifacts to make the findings of this study applicable to the context of an automated resilience intervention that does not rely on human interference during data management. In addition, the use of single-item scales in the evening EMA questionnaire forms a limitation of comprehensiveness at which the concepts can be measured. Therefore, validated single-item scales or items with the most favorable psychometric properties in existing validated scales were used where available [39]. As single-item scales have consistently been found to be valid measures for diverse concepts in comparison to full scales [81-84] and have become common good in EMA research, the applied EMA methods can still be considered appropriate. The participants also received some feedback on their sleep, physical activity, and HRV because of the use of the consumer-available Fitbit and Elite HRV apps, which might have influenced their behavior. Nevertheless, any such influence was not considered a problem because this study observes the natural relationship between several variables and does not reflect on the behaviors themselves. Finally, only 3.6% of the within-subject variance in resting HRV could be explained, and the buffering effect of resting HRV was relatively modest.

### Generalizability

The HRV-related results can be generalized to young and employed female adults who track their resting HRV upon awakening. As 92% (24/26) of the participants were female and HRV can be related to menstrual cycle changes [85], further research on young males is necessary to improve the generalizability of these findings to young adults regardless of gender. As the resting HRV was measured upon awakening in this study, the influence of a phenomenon called the cortisol awakening response (CAR) might have played a role. Upon awakening, cortisol levels start to increase and peak approximately 30-45 minutes thereafter because of the CAR, where 1%-3.6% of its variance can be explained by psychosocial factors [86]. Although the CAR is associated with postawakening changes in HRV, these changes appear to be unrelated to perceived stress and measures of emotion regulation [87]. Therefore, it is possible that measuring HRV during sleep could yield similar results. An advantage of measuring resting HRV during sleep is that participants would not need to apply a moistened chest strap and lay still in a supine position upon awakening to collect their resting HRV data. As multiple participants described that this procedure negatively impacted their adherence to the measurement protocol, unobtrusively measuring the resting HRV during sleep might improve adherence and thus increase statistical power. Future research is needed to confirm whether resting HRV during sleep can be used to yield similar results.

### Implications

To our knowledge, this study is the first to report a significant within-subject buffering effect of resting HRV on the positive associations between demands and stress, as well as between stress and mental exhaustion and a negative association between mental exhaustion and resting HRV. Replication of these findings in future studies is needed. As the combined findings form a feedback loop, it is possible that multi-day trends in resting HRV could be linked to longitudinal mental health outcomes in future studies. Furthermore, exploring the use of time series analysis to create within-subject models in which multi-day trend data are used to assess the daily outcomes could potentially improve the accuracy of the presented models.

Future studies are advised to use passive monitoring techniques that require little to no user attention whenever possible to improve participant adherence and optimize statistical power.

If the findings of this study can indeed be replicated and expanded upon, it would show that longitudinally monitoring resting HRV as a biomarker of or index for resilience may be useful in the context of prevention. In this context, a structural increase or decline in resting HRV could provide an early warning signal that a positive or negative feedback loop is formed. When used in a consumer wearable–based automated resilience intervention, these signals can be used to prompt user feedback. For instance, users could be rewarded when a positive feedback loop is recognized or suggested to perform cognitive behavioral therapy–based self-reflection exercises or relaxation techniques when a negative feedback loop occurs. Such an automated resilience intervention that unobtrusively monitors the user’s resting HRV for the early recognition of (un)favorable feedback loops and generation of just-in-time feedback may therefore limit the buildup of allostatic load and improve long-term health outcomes.

## Abbreviations

CAR: cortisol awakening response
EMA: ecological momentary assessment
HRV: heart rate variability
MVPA: moderate-to-vigorous physical activity
RMSSD: Root Mean Square of the Successive Differences
TST: total sleep time
